# Reconsidering Gender in Asthma: Is It All About Sex? A Perspective Review

**DOI:** 10.3390/jcm14072506

**Published:** 2025-04-07

**Authors:** Alessio Marinelli, Silvano Dragonieri, Andrea Portacci, Vitaliano Nicola Quaranta, Giovanna Elisiana Carpagnano

**Affiliations:** Department of Respiratory Diseases, University of Bari, 70121 Bari, Italy; alessio.marinelli@uniba.it (A.M.); a.portacci01@gmail.com (A.P.); vitalianonicola.40@gmail.com (V.N.Q.); elisiana.carpagnano@uniba.it (G.E.C.)

**Keywords:** asthma, gender, exacerbation, severity, perspective, review

## Abstract

Asthma is a prevalent chronic condition, affecting an estimated 260 million people worldwide, according to the 2021 Global Burden of Disease Study. This condition significantly impacts individuals of all ages. One notable finding is that asthma prevalence among adults was higher in females than males. Recent evidence suggests that these disparities in asthma prevalence and outcomes are likely due to complex interactions among hormonal, anatomical, and environmental factors, coupled with societal and behavioral influences. The interchangeable use of the terms “sex” and “gender” in the scientific literature is frequently inconsistent. Biological sex is defined by anatomical and physiological characteristics determined by genetics; “gender”, on the other hand, is a more complex construct and a universally accepted definition is still lacking. This lack of clarity, coupled with potential knowledge gaps, misunderstandings, or the inherent difficulty in differentiating sex- and gender-related effects, often leads to the terms being poorly defined or used interchangeably. Such imprecise usage hinders accurate data interpretation and research progress. This paper provides a perspective review synthesizing current knowledge regarding the influence of sex and gender on asthma, specifically focusing on their impact on disease pathogenesis, clinical presentation, severity, and management strategies.

## 1. Introduction

Asthma is a prevalent chronic condition affecting millions worldwide. According to the Global Burden of Disease Study, an estimated 260 million people were living with asthma in 2021 [1]. This condition significantly impacts individuals of all ages, leading to premature death and a diminished quality of life. Globally, asthma ranks as the 24th-leading cause of years lived with disability and the 34th-leading cause of overall disease burden [2]. Asthma imposes a substantial economic burden globally due to both direct and indirect costs. While age-specific asthma mortality rates have decreased in several countries, the total number of asthma-related deaths has remained relatively stable due to population aging. Avoidable asthma fatalities continue to occur due to inadequate management, often involving over-reliance on quick-relief medications instead of preventive therapies. The Global Burden of Disease Study (GBD) [1], a comprehensive global health research initiative, has made significant contributions to understanding the epidemiological burden of diseases worldwide. By analyzing data from 1990 to 2021 across 244 countries, GBD has revealed important trends in asthma prevalence. One notable finding is that asthma prevalence among children was higher in males than females, but this pattern reversed in adulthood. Similar trends were observed in disability-adjusted life years (DALY), a measure of the overall health impact of diseases and injuries, and incidence rates. However, mortality rates for asthma were consistently higher in females across all age groups.

Generally, women outlive men, but their healthy life expectancy is comparable, particularly in low-income countries. The gap in both life and healthy life expectancy narrows after the age of 60. A gender-based approach to clinical practice can promote tailored care that reflects the increasing healthy life expectancy observed in high-income countries [3]. Historically, medicine has exhibited an androcentric bias, with limited attention to women’s health beyond reproductive issues. Only recently have gender and sex differences been fully recognized in the medical field according to The World Health Organization (WHO) definition of gender medicine, described as the study of the effects of influences of both sex and gender on health.

Emerging evidence suggests significant differences in the development, progression, and clinical manifestations of conditions that affect both men and women. These differences also extend to adverse events associated with treatments, responses to therapies and nutrients, and lifestyle factors [4].

Recent evidence suggests that these disparities in asthma prevalence and outcomes may be rooted in complex interactions among hormonal, anatomical, and environmental factors, coupled with societal and behavioral influences. While the exact reasons for these differences remain to be fully elucidated, one potential explanation lies in anatomical variations, as suggested in 1980 by Mead [5]: adult females show smaller airways compared to males with similar lung volumes. This phenomenon is called dysanapsis, and it could contribute to increased airway resistance and more severe airway hyperresponsiveness (AHR) found in females. So it may appear to be merely a sex question.

Nevertheless, asthma is a heterogeneous condition, characterized by varying degrees of severity, natural history, and treatment responsiveness. The interplay between sex hormones, airway inflammation type, and exacerbations is a complex area that warrants further investigation.

Historically, asthma management has been based around the concept of phenotypes: a combination of clinical and biological traits. However, recent advancements have shifted the focus towards associating molecular mechanisms with these phenotypes, leading to the concept of asthma endotypes.

The T2-high endotype, observed in both pediatric and adult populations, is typically associated with eosinophilic inflammation, where eosinophils play a crucial role in perpetuating type 2 inflammation in asthmatic patients. Early-onset or extrinsic allergic asthma is a classic example of the T2-high asthma phenotype, often affecting male patients with atopy and a history of sensitization to inhalant allergens. On the other hand, T2-low disease is often linked to neutrophilic or paucigranulocytic inflammation and poor response to corticosteroid therapy. Females with severe late-onset asthma often fall under the T2-low endotype, particularly when neutrophilic inflammation is present [6].

Sex hormones also likely play a role in the increased exacerbation risk observed in female patients: evidence suggests that airway hyperresponsiveness is more pronounced during the luteal phase of the menstrual cycle and that pregnancy increases the likelihood of exacerbations. Conversely, androgens, such as testosterone, may have a protective effect against asthma by reducing incidence and symptoms [7].

In addition to hormonal effects, environmental factors and exposures could contribute to the sex and gender differences. Factors such as socioeconomic level, geographical area, immunization records, the number of pregnancies, breastfeeding habits, and exposure to secondhand smoke and other contaminants could alter the equilibrium between T2 and non-T2 inflammation [8,9,10,11,12].

The interchangeable use of the terms “sex” and “gender” in the scientific literature is frequently inconsistent. Biological sex is commonly defined as a binary trait defined by anatomical and physiological characteristics determined by genetics; “gender”, on the other hand, is a more complex construct, reflecting culturally defined roles, behaviors, and identity presentations among various groups of people. Notably, a universally accepted definition of “gender” is still lacking. This lack of clarity, coupled with potential knowledge gaps, misunderstandings, or the inherent difficulty in differentiating sex- and gender-related effects, often leads to the terms being poorly defined or used interchangeably. Such imprecise usage hinders accurate data interpretation and research progress. A perspective review was undertaken to synthesize the current knowledge regarding the influence of sex and gender differences on asthma, specifically focusing on their impact on disease pathogenesis, clinical presentation, severity, and management strategies.

## 2. Materials and Methods

Our study was conducted in three distinct phases.

### 2.1. Phase 1: Epidemiological Overview

In this phase, we conducted a comprehensive search to obtain the most reliable and up-to-date epidemiological data on asthma, aiming to understand the global prevalence and distribution of the disease. We prioritized open-access, fully accessible, and globally representative data sources.

Unfortunately, we did not find an institutional database, such as a WHO disease database. Therefore, we utilized the Global Burden of Disease Study database [1], an extensive research initiative based at the Institute for Health Metrics and Evaluation (IHME) at the University of Washington. This database, involving a vast global consortium of researchers, systematically analyzes the health status of populations across diverse regions worldwide. It offers fully open access to its data, which was updated to 2021.

### 2.2. Phase 2: Global Literature Review

We conducted a literature search in the PubMed and Google Scholar databases to gain a comprehensive understanding of the research literature on gender and sex differences in asthma. This phase aimed to assess how these differences are considered in the research context.

For PubMed/Medline, we used the search syntax “(gender OR sex) AND (child OR childhood OR adolescence OR adult) AND (asthma)”. The search was restricted to articles in English with available full texts. We prioritized the most recent articles, limiting the initial screening to studies published from 2019. Two researchers (AM, SD) independently screened the titles and abstracts of all retrieved articles, prioritizing those published in Q1 journals. In cases of disagreement, consensus on which articles to screen in full text was reached through discussion.

For Google Scholar, we used the search syntax “gender sex asthma”. The search was restricted to articles in English with available full texts. We prioritized the most recent articles, limiting the initial screening to studies published from 2019 with the highest citation scores. The same two researchers independently screened the titles and abstracts of all retrieved articles. In cases of disagreement, consensus on which articles to screen in full text was reached through discussion.

Subsequently, duplicates were removed. Finally, the two researchers independently screened the full-text articles for inclusion. Again, in cases of disagreement, consensus on inclusion or exclusion was reached through discussion.

### 2.3. Phase 3: Local Insight

After obtaining a global perspective on sex and gender differences in asthma, we identified four major points of discussion that required further exploration to enhance our understanding:

- Anatomical Factors;
- Hormonal Factors;
- Environmental Factors;
- Lifestyle Factors.

As in the previous phase, we conducted searches in the PubMed and Google Scholar databases for each of the four points.

For PubMed, we added each point as a new keyword to the previous search strings. The search was restricted to articles in English with available full texts. Two researchers (AM, SD) independently screened the titles and abstracts of all retrieved articles, prioritizing those published in Q1 journals. In cases of disagreement, consensus on which articles to screen in full text was reached through discussion.

For Google Scholar, we added each point as a new keyword to the previous search strings. The search was restricted to articles in English with available full texts. We prioritized articles with the highest citation scores. The same two researchers independently screened the titles and abstracts of all retrieved articles. In cases of disagreement, consensus on which articles to screen in full text was reached through discussion.

We did not exclude studies based on the date of publication, as we aimed to include older papers that could provide a starting point for discussing the relevant questions.

Subsequently, duplicates were removed. Finally, the two researchers independently screened the full-text articles for inclusion. Again, in cases of disagreement, consensus on inclusion or exclusion was reached through discussion.

## 3. Revisiting Gender Disparities in Asthma

### 3.1. Biological Influences: Anatomical Factors

The embryonal development of the respiratory tract, characterized by complete airway differentiation and branching patterns, concludes by the 16th gestational week [13]. Notably, no sexual dimorphism in this developmental process has been reported. However, at birth, females showed smaller lungs compared to males [8]. In the passage from childhood to adulthood, during adolescence, sexual dimorphism is inverted: females have larger lungs, as they are taller when compared to their male counterparts because of pubertal growth spurt [14]. In adulthood, instead, men have bigger lungs and show greater mean values for all pulmonary variables (flow and volume measurements), except airway resistance, which is significantly lower [15]. The number of alveoli per unit area and alveolar dimensions do not differ between males and females, but bigger lungs per unit of stature mean a larger total number of alveoli and a larger alveolar surface area for a given age and stature [13].

**Figure 1:**
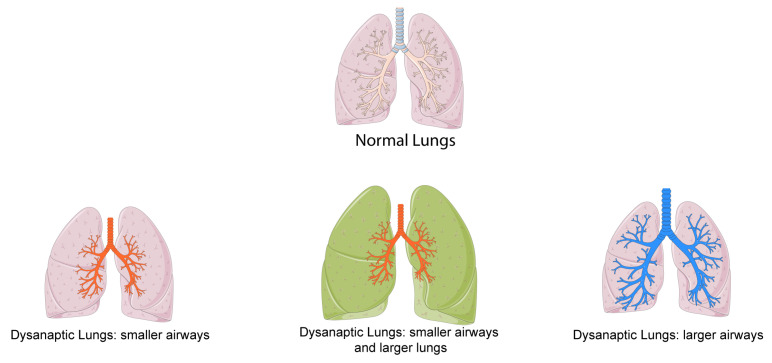
Representation of lung dysanapsis. Lung and trachea–bronchi icons by Servier (https://smart.servier.com/) are licensed under CC-BY 3.0 Unported (https://creativecommons.org/licenses/by/3.0/, accessed on 28 March 2025).

Green’s work in 1974 [16] first posited that equivalent lung volumes do not necessarily correlate with identical airway diameters (Figure 1). This disparity, termed “dysanapsis”, accounts for a significant portion of inter-individual variability in expiratory flow rates. Building upon this concept, Mead’s research [5] revealed distinct sexual dimorphism, with female airways being disproportionately smaller relative to lung size compared to males; these differences develop late in growth.

Dysanapsis, defined by Mead [5] as the ratio of maximal expiratory flow divided by static recoil pressure at 50% of vital capacity, provides an index of the structure–function relationship between airway size and lung size. Utilizing flow dynamics models, Mead [5] estimated that male airways possess a diameter approximately 17% greater than female airways, suggesting a late-stage developmental divergence. This observation aligns with subsequent findings from acoustic reflection studies of the trachea [17] and computed tomography imaging [18]. However, no difference in lung intrinsic elasticity between sexes has been reported [19].

In 2015, Dominelli et al. [20], employing high-resolution computed tomography, revisited the dysanapsis concept, confirming that, in lung size-matched cohorts, female subjects exhibit significantly reduced luminal areas in larger conducting airways [18]. Given that larger airways constitute the primary site of respiratory resistance and smaller airways contribute only a minor fraction, female patients exhibited increased airway resistance, particularly during high ventilatory demand, such as exercise. Their study uniquely integrated anatomical assessments with detailed lung mechanics during exercise, demonstrating a substantially elevated total work of breathing ($W_{b}$) in female patients relative to males for a given ventilation rate (${\dot{V}}_{E}$). They also confirmed no sex differences in the intrinsic elasticity of lungs, as the increased ($W_{b}$) is due to a higher resistive work ($W_{b,r}$) rather than viscoelastic work ($W_{b,v}$). A reduced dysanapsis ratio, indicating narrower airways relative to lung volume, was associated with mechanical ventilatory constraints during exercise, particularly in individuals with high aerobic capacity, regardless of sex. These results indicate that inherent sex-based differences in airway morphology lead to significant disparities in $W_{b}$ during exercise. While $W_{b}$ did not differ between sexes at rest or low ${\dot{V}}_{E}$, it increased disproportionately in women at higher ${\dot{V}}_{E}$, primarily due to the work required to overcome turbulent flow resistance. To further increased the disparities in $W_{b}$ during exercise, women, especially the healthy fit ones, show a relative hyperinflation at higher ${\dot{V}}_{E}$. Due to the reduced diameter of airways and smaller lung volumes, females are more susceptible to developing expiratory flow limitation (EFL) compared to males. EFL is a mechanical pathophysiological condition that manifests during both physical activity and rest. The expiratory flow cannot be further increased by greater effort from the expiratory muscles, as it is already maximal at the given tidal volume (V_D_). Higher expiratory flows can only be achieved by progressively shifting the end-expiratory lung volume (EELV) towards total lung capacity (TLC) with a concomitant increase in functional residual capacity (FRC). All these changes promote the onset of dynamic hyperinflation (DH). DH results in the development of a positive end-expiratory alveolar pressure (PEEP_i_), which contributes to an increased $W_{b}$. When DH becomes pronounced, it leads to significant neuro-mechanical dissociation, a pathological state where high inspiratory effort (due to increased elastic load and less efficient inspiratory muscles) is associated with a reduced and insufficient increase in lung and chest wall volume [21].

In 2022, the American Thoracic Society/European Respiratory Society “Technical standard on interpretive strategies for routine lung function tests” document [22] defines dysanapsis a spirometric pattern characterized by “a low FEV_1_/FVC ratio accompanied by FEV_1_ within the normal range […] due to dysanaptic or unequal growth of the airways and lung parenchyma”.

Dos Santos et al. [23] first described the factors associated with this pattern in healthy individuals, including male sex, younger age, and taller stature, along with higher forced vital capacity (FVC) above predicted values and elevated expiratory flows as indicated by forced expiratory flow at 75% (FEF_75%_). However, the same researchers noted that the normal variant group consisted predominantly of males, suggesting that dysanapsis, as originally defined by Mead [5], was not responsible for the low FEV_1_/FVC ratio. Instead, they proposed that inverse effort dependence might be a plausible explanation. In adults, the FEV_1_/FVC ratio decreases with increased stature in both sexes because the greater length of repiratory muscles results in greater force, potentially leading to decreased FEV_1_ due to inverse effort dependence [24]. FEV_1_ is inversely dependent on effort due to thoracic gas compression, which is affected by alveolar pressure and lung volume. This is a universal phenomenon, particularly true in young and tall males, as they generally exhibit greater muscular force and higher FVC values. However, flows at low lung volumes are less influenced by respiratory effort, and a reduction can be observed early in obstructive lung diseases, making them a possible spirometric marker to distinguish normal variants from obstructive defects.

### 3.2. Biological Influences: Hormonal Factors

In females, one of the two X chromosomes undergoes random inactivation to balance gene expression levels between the sexes. This process, known as X chromosome inactivation (XCI), leads to cellular mosaicism, where approximately half of the cells in a tissue express genes from the maternal X chromosome and the other half from the paternal X chromosome. XCI is established early in female embryogenesis; however, 15–23% of X-linked genes escape this inactivation and are expressed from both the active (Xa) and inactive (Xi) X chromosomes in certain tissues or individuals [25,26]. The differences between male and female immune systems are influenced by biological factors such as sex steroid hormones produced by the ovaries and testes, which act through nuclear receptor family members. These hormones and sex chromosomes can work together or independently to directly or indirectly regulate various factors within host immune cells. This regulation occurs through cell-intrinsic signaling or the sex-specific control of X-linked gene expression, thereby directly affecting immune cell development and function [27]. Sex hormones exert their effects via estrogen receptors (ERα and ERβ), androgen receptor (AR), and progesterone receptors (PRα and PRβ), which are ligand-inducible transcription factors. These receptors regulate gene transcription by binding directly to specific response elements on DNA, leading to epigenetic modifications of chromatin and the direct transcription of target genes. Immune cells, including those of the innate immune system, express sex-steroid hormone receptors such as ERs and AR to varying degrees, depending on their microenvironment [28].

**Figure 2:**
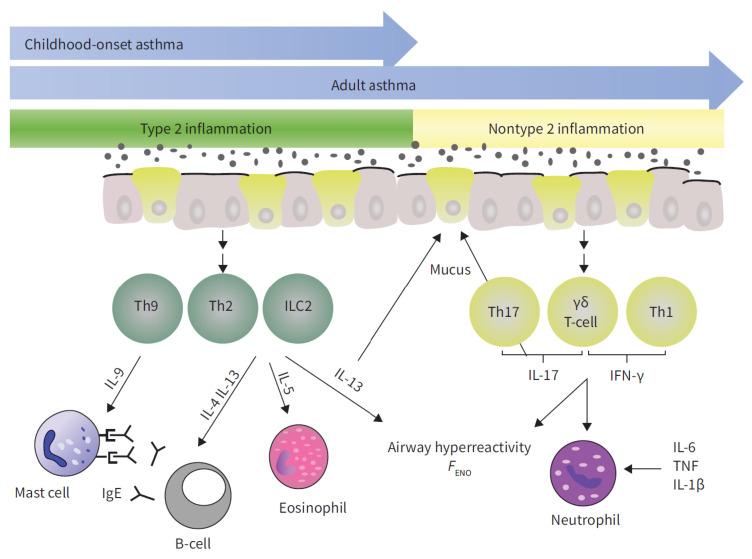
A schematic representation of inflammatory pathways in asthma: T2 inflammation is reported in green, non-T2 inflammation in yellow. AHR: airway hyperresponsiveness; FENO: exhaled nitric oxide fraction; IFN: interferon; IL: interleukin; ILC2: type 2 innate lymphoid cell; Th: T-helper cell; TNF: tumour necrosis factor; TSLP: thymic stromal lymphopoietin. Image taken from [7].

Asthma’s immune mechanisms are classified into two primary endotypes (Figure 2): the T2-high endotype and the T2-low endotype. The T2-high endotype is characterized by predominant type 2 (T2) inflammation, which involves the production of interleukins (IL) such as IL-4, IL-5, IL-13, and IL-9 by type 2 innate lymphoid cells (ILC2s) and/or CD4+ T-helper type 2 cells (Th2). This increased production of type 2 cytokines results in the elevated levels of IgE, activation of mast cells, production of mucus and exhaled nitric oxide fraction (FENO), eosinophil infiltration and activation, and airway hyperresponsiveness (AHR). In contrast, the T2-low endotype is characterized by predominant non-T2 inflammation, which involves neutrophilic or paucigranulocytic infiltration, mucus production, steroid resistance, and AHR. This endotype is mediated by the increased production of interferon gamma (IFN-γ), tumor necrosis factor (TNF-α), and IL-17A from Th17 cells [6,7,29].

Research using mice has been fundamental in uncovering the cellular mechanisms underlying asthma pathogenesis and the influence of sex chromosomes and hormones [27,30]. For instance, studies involving castration and hormone replacement in mice have demonstrated that the castration of male mice eliminates the sex bias in asthma. The administration of 5α-dihydrotestosterone (DHT) or dehydroepiandrosterone (DHEA), a natural steroid hormone produced by the adrenal gland and converted into androgens or estrogens, mitigates ovalbumin (OVA)-induced asthma [27]. Additionally, in a mouse model with a mutation in the androgen receptor (AR) gene, rendering the mice unresponsive to androgens, it has been shown that AR signaling reduces house dust mite (HDM)-associated eosinophilia [31]. Furthermore, in an OVA-induced asthma mouse model, ovarian hormones exacerbate lung inflammation, as female mice exhibit higher levels of eosinophils, effector T cells, and M2-polarized macrophages in the lungs compared to males [32].

Estrogen and progesterone significantly impact immune responses, promoting either eosinophilic or neutrophilic inflammation. Estrogen receptors (ERs) and progesterone receptors (PRs) are present in human T cells, and their activation by estradiol and/or progesterone enhances the production of IL-4 and IL-17 [30]. On the other hand, androgens may contribute to protective immune responses against lung inflammation. Testosterone and other androgens, through signaling via androgen receptors (ARs), reduce the proliferation of type 2 innate lymphoid cells (ILC2), eosinophil infiltration, and the production of IL-33, thymic stromal lymphopoietin (TSLP), and type 2 cytokines [33].

The reactivation of immune-related genes linked to the X chromosome may contribute to sex-dependent immune responses and disease progression. Similarly, asthma has been observed in male patients with Klinefelter’s syndrome, where the genetic influence of the X chromosome, coupled with the negative impact of relatively lower testosterone levels, is implicated in asthma pathogenesis [34]. Therefore, while female sex and sex chromosomes are linked to asthma pathogenesis, the detailed mechanisms driving the severity of asthma in women are not yet fully understood.

Approximately 20% of women demonstrate a correlation between the premenstrual phase and asthma severity [35]. This population is commonly characterized by older age, elevated BMI, a longer history of asthma, and a higher prevalence of aspirin-exacerbated respiratory disease. They also exhibit a greater likelihood of experiencing dysmenorrhea, premenstrual syndrome, shortened menstrual cycles, and extended menses. Hanley et al. [36] first documented in 1981 that asthmatic women exhibit worsened symptoms during menstruation, characterized by increased airway resistance rather than purely psychological factors. Subsequent research, including a systematic review by Sanchez et al. [37], has investigated the cyclical deterioration of asthma during the premenstrual or perimenstrual phase, reported as “premenstrual asthma” (PMA). While the perimenstrual phase is the most frequently studied, this deterioration can also occur during the periovulatory, mid-preovulatory, or luteal phases. The pathophysiology connecting sex hormones to premenstrual asthma exacerbation is thought to involve the modulation of airway inflammation.

In a systematic review, McCleary et al. [38] investigated the relationship between puberty timing, menstrual characteristics, menopause, and the risk of developing asthma. The study found that early puberty was associated with a higher risk of asthma, while delayed puberty seemed to offer some protection. Early menarche, defined as the onset of menstruation before the age of 11 years, was associated with an increased likelihood of both new and ever asthma diagnoses compared to typical menarche, occurring between the ages of 11 and 13 years. Late menarche, defined as onset after 13 years, was associated with asthma, but not new-onset cases. Irregular menstruation, often defined by self-report or cycle length >32 days, was associated with increased current asthma risk, particularly atopic asthma, but not current wheeze or non-atopic asthma. Menopause onset was linked to increased current asthma risk and variably associated with new-onset asthma across studies. Menopause was also associated with increased current wheeze risk. Exacerbation rates and asthma medication use appeared highest around menopause, although statistical significance was not always reported.

Observational studies involving premenopausal women have demonstrated a potential association between oral contraceptive pill (OCP) utilization and a reduction in asthma incidence, alongside a corresponding decrease in asthma-related healthcare resource utilization. This effect appears to be particularly pronounced in lean women [39]. Furthermore, evidence suggests that OCPs may mitigate wheezing symptoms in individuals with asthma [40]. Recent analyses of the Optimum Patient Care Research Database have corroborated these findings, indicating an inverse relationship between hormonal contraceptive use, the duration of use, and the risk of asthma incidence [41,42]. Collectively, these studies suggest a potential protective effect of hormonal contraceptives against asthma incidence and symptom severity. However, further research is imperative to delineate the specific hormonal contraceptive formulations that confer optimal benefits in terms of asthma risk reduction and symptom management.

The influence of hormone replacement therapy (HRT) administered during the menopausal transition on both the likelihood of asthma development and the management of existing asthma has been investigated extensively. Notably, analyses conducted utilizing the Optimum Patient Care Research Database have revealed a correlation between the utilization of HRT and a diminished probability of de novo asthma diagnosis. Furthermore, these analyses suggest a dose-dependent relationship, wherein prolonged HRT usage appears to correlate with a proportional decrease in asthma incidence. Similarly, the longitudinal U.S. Nurses’ Health Study (NHS) cohort demonstrated a discernible reduction in asthma incidence among premenopausal women compared with postmenopausal women, as documented by Troisi et al. [43]. Reinforcing this, the Severe Asthma Research Program (SARP) study indicated that, among participants aged 45 years and older, severe asthma was more prevalent in males than in females, a reversal of the trend observed in younger adult populations, as reported by Zein et al. [44]. Further elucidating the complex interplay between hormonal status and asthma, Scioscia et al. [45] found that an increased asthma severity was associated with statistically significant elevations in circulating 17β-oestradiol levels among female patients with postmenopausal asthma. However, the precise nature of the relationship between menopause and asthma remains ambiguous. While some women experience asthma onset coincident with the menopausal transition, other factors, such as the increased prevalence of comorbid conditions and HRT use during menopause, confound the elucidation of this association.

Notably, a Phase II clinical trial [46] showed that the use of exogenous androgens (nebulised DHEAS) could improve asthma symptoms in female patients with moderate or severe asthma: there was a reduction in Asthma Control Questionnaire (ACQ) scores when compared with those who received placebo. Further, another study [47] suggested that exogenous androgens may improve FEV1 in premenopausal female patients with mild or moderate asthmatic and low baseline DHEA-S levels (<200 μg/dL) compared to those with baseline DHEA-S ≥ 200 μg/dL.

Several studies have investigated the impact of pregnancy on asthma severity and symptom presentation. Schatz et al. [48] observed that approximately one-third of pregnant asthmatics, monitored through daily symptom diaries and monthly spirometry, experienced an increase in asthma symptoms. Notably, the majority (73%) of these women saw their symptoms return to pre-pregnancy levels within three months postpartum. Yung et al. [49] reported that women with severe asthma exhibited a higher risk of exacerbations during pregnancy, primarily triggered by viral infections and poor adherence to inhaled corticosteroid regimens. This highlights the importance of medication adherence in this population. Conversely, Juniper et al. [50] found a decrease in airway responsiveness and asthma severity (measured by FEV1/vital capacity percentage) during pregnancy in a cohort of 16 women, with a return to pre-conception levels one month postpartum. This suggests that, in some individuals, pregnancy may have a transient attenuating effect on certain asthma parameters. Furthermore, Belanger et al. [51] concluded that pregnancy did not influence asthma severity, provided that prescribed medications were consistently used. Their research also indicated no correlation between pregnancy trimester and changes in asthma status.

### 3.3. Environmental Factors

While environmental factors, such as airborne pollutants, affect both sexes, the prevalence of exposure to specific substances, recognized as risk factors for asthma and respiratory illnesses, differs between women and men due to their respective societal and occupational roles [52]: women are more often exposed to cleaning solutions and fuels derived from organic sources, while men have a higher incidence of exposure to products resulting from thermal decomposition, vegetable matter, isocyanates, and metallic substances [53]. However, evolving gender roles necessitate ongoing investigation into potential exposure disparities in occupations traditionally associated with a single gender. Shifting work and home dynamics may ultimately mitigate gender-based differences in exposure to asthma triggers.

### 3.4. Lifestyle Factors

Women frequently report more pronounced asthma symptoms than men, including increased frequency, severity, and bothersomeness, even when objective measures of asthma control and severity are comparable [54]. This disparity extends to their quality of life, with women reporting greater symptom impact and limitations on physical activities, social engagements, sleep, and daily routines. Consequently, women are more likely to consult healthcare providers (HCPs) regarding their asthma [55]. Conversely, societal expectations regarding masculinity may contribute to symptom underreporting and the reluctance to seek professional medical attention in men [53].

Other lifestyle factors also play a role. While men and boys typically engage in more regular physical activity and exhibit lower rates of obesity [56], potentially mitigating asthma symptom risk, dietary habits may present a counterpoint.

Research suggests that men exhibiting strong “gender-conforming” traits may adhere to less healthy diets compared to other groups [57]. Given the established efficacy of physical activity and healthy dietary patterns in improving asthma outcomes for both sexes [58,59], promoting these interventions, which could potentially positively influence asthma risk and management, is crucial. Motivational factors should also be considered. Men often cite competition, health maintenance, and body image as primary motivators for physical activity, while women are more frequently driven by social support, emotional wellbeing, and positive body image [60].

Smoking prevalence is higher in men [61], but women appear more susceptible to smoking-related asthma symptoms [62]. Furthermore, women face greater challenges in smoking cessation due to increased susceptibility to nicotine addiction. This difference is likely attributable to faster nicotine metabolism in women, resulting in a higher nicotine requirement to achieve desired effects [63].

Finally, the higher prevalence of comorbid anxiety in women with asthma [53] warrants consideration. Because anxiety and stress can sometimes lead healthcare providers (HCPs) to underestimating patient symptom severity, women with comorbid anxiety may be at increased risk for misdiagnosis and suboptimal asthma management [53].

## 4. Discussion

In our review, we identified a significant limitation in retrieving data on asthma epidemiology. We have not found a fully open-access database provided by a public institution, and it is our opinion that this represents a serious impediment to researchers. It would be advisable to establish a public registry, potentially under the auspices of the World Health Organization (WHO) or a similar institution, to provide a free and unbiased database of diseases. Currently, we rely on the Global Burden of Disease, which is an exceptional effort provided by the University of Washington to address this limitation. Despite the data being updated only until 2021, we believe it remains a reliable source.

**Figure 3:**
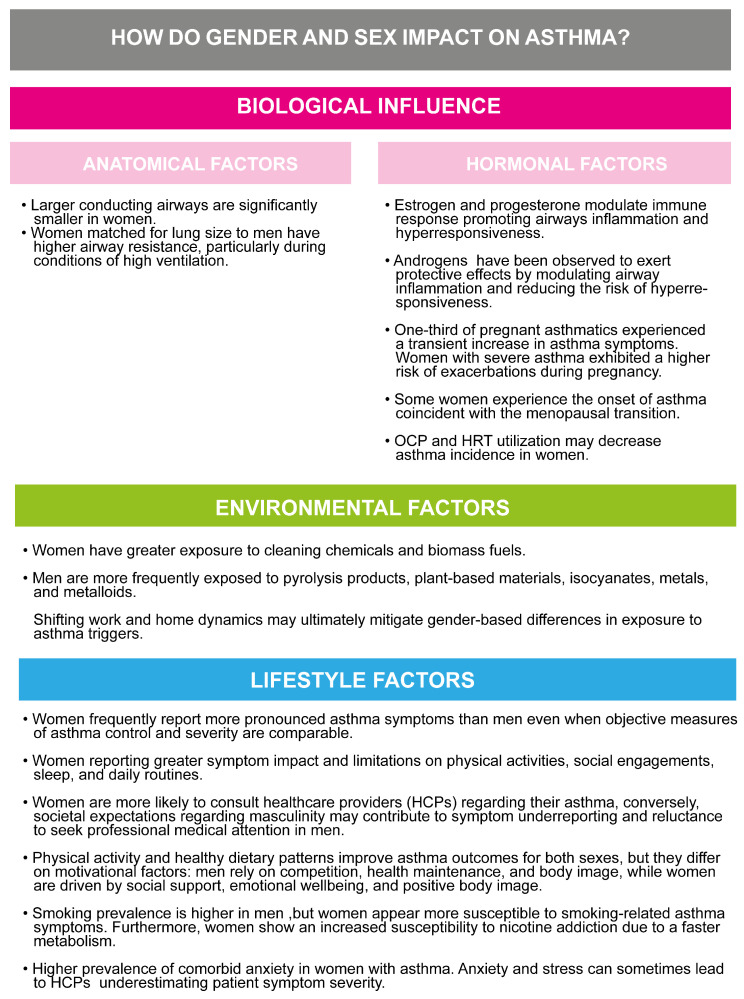
Summary of sex- and gender-specific factors affecting asthma, including biological, environmental, and lifestyle factors, and their impact on disease outcomes.

Next, we found the scientific literature on gender and sex differences in asthma to be substantial, reflecting how it is a hot topic in current research. Nonetheless, current guidelines often adopt a one-size-fits-all approach, which may not address the unique needs of individuals, despite emerging evidence, as resumed and reported in Figure 3.

One major challenge is the role of hormonal influences in asthma severity. While estrogen and progesterone appear to worsen asthma severity, androgens may offer a protective effect. Estrogen and progesterone are known to modulate airway inflammation and hyperresponsiveness, influencing immune responses and promoting either eosinophilic or neutrophilic inflammation. Androgens, on the other hand, have been observed to exert protective effects by modulating airway inflammation and reducing the risk of hyperresponsiveness. However, the precise mechanisms remain an area of active research.

Another challenge is the limited availability of high-quality data, particularly from studies involving diverse populations. Transgender patients receiving consistent hormone therapy provide a unique model for examining the impact of hormones on asthma symptoms and severity, as exogenous sex hormone administration may mimic endogenous hormonal effects. Nonetheless, there is a lack of extensive research on asthma within this population. We report the study by Zein et al. [64], which included 7210 patients with gender dysphoria and 490 post-gender-affirming surgery, showing the highest asthma risk in male-to-female transgender individuals, with a statistically significant increase also observed in female-to-male transgender individuals. Those findings raised questions about the previously held belief that exogenous and endogenous sex hormones have equivalent effects, and raised critical questions regarding the potential respiratory consequences associated with cross-sex hormone therapy.

Conversely, there are several studies on cisgender population. We found a well-documented body of literature about hormonal influences in females. The observation that asthma symptoms worsen during specific phases of the menstrual cycle in a significant proportion of women underscores the need for personalized management strategies. Similarly, premenopausal and postmenopausal women may benefit from treatment strategies that address hormone-related changes in airway inflammation and responsiveness. For instance, asthma management during pregnancy requires careful monitoring and adjustments to minimize risks to both the mother and fetus. The influence of hormonal contraceptives and hormone replacement therapy on asthma risk and severity should lead to a multidisciplinary approach between pulmonologists, endocrinologists, and gynecologists to develop a personalized management plan that account for hormonal fluctuations.

Environmental factors also play a crucial role. Occupational exposures, particularly in traditionally gendered professions, can significantly impact asthma development and control. Differences in lifestyle factors, including tobacco use, dietary habits, or exercise levels, may increase gender-based disparities in asthma. Women, for instance, may face unique challenges in smoking cessation due to differences in nicotine metabolism. Additionally, the interplay between diet, physical activity, and asthma control may be influenced by gender-specific motivational factors. As societal roles evolve a deeper understanding of how these exposures affect individuals of different genders is essential.

In our opinion, we should expect a future with less pronounced gender differences in lifestyle factors and environmental exposure; it will become crucial to investigate the sex-related differences, particularly the hormonal influence.

## 5. Conclusions

The clinical progression of asthma is demonstrably affected by both biological sex, delineated as male or female, and gender, a complex social construct involving roles, behaviors, and self-expression.

The observed differential prevalence of asthma across the lifespan, with a higher incidence in boys prior to puberty and subsequently in women post-puberty, may be attributed to variations in pulmonary physiological development and the modulatory effects of sex hormones. The ongoing influence of female sex hormones contributes to poorer asthma control. Furthermore, gender-specific factors may result in varying exposure to asthma triggers, and both sex and gender can impact the frequency of comorbidities and the dynamics of healthcare interactions. Although these disparities in asthma are now well-established, these crucial factors have often been ignored in clinical practice.

First, we believe it is crucial to establish a worldwide registry of diseases, modeled after the Global Burden of Disease (GBD) but under the control of an international public institution to guarantee impartiality. This registry should be fully open access and actively involve all researchers and institutions worldwide. This would be the first step to ensuring high-quality data, especially regarding the epidemiology of diseases.

Next, healthcare providers (HCPs) need to change the conventional, monolithic approach to asthma care and embrace a paradigm that acknowledges and addresses the unique needs of each individual, with due consideration to their sex and gender. This should also be reflected in international guidelines, as a personalized approach is essential for optimizing therapeutic outcomes and enhancing the quality of life for individuals afflicted with asthma.

When performing pulmonary function tests (PFTs), HCPs should always be aware of dysanapsis. A low FEV_1_/FVC ratio with a preserved FEV_1_ should not be addressed as obstructive, but, if expiratory flows at low lung volumes are normal, they may indicate a dysanaptic pattern that is a physiological variant, especially in young and tall males.

This new paradigm also requires that HCPs consider not only the sex but also the gender identity of their patients in the future, to achieve a truly tailored approach to asthma.

Pulmonologists and other physicians involved in asthma care should seek multidisciplinary consultations, particularly with endocrinologists and/or gynecologists, especially for patients with “difficult-to-treat” asthma. For example, women experiencing asthma exacerbations during the luteal phase may benefit from targeted interventions, such as the use of oral contraceptive therapy. Additionally, the use of leukotriene receptor antagonists in women should be limited, at least until the presence of comorbid anxiety has been verified.

Finally, we posit that an urgent and concerted effort is warranted to prioritize gender-specific research and extend its scope to encompass a broader demographic, including not only cisgender individuals but also transgender patients.

By prioritizing such research endeavors and implementing personalized therapeutic strategies, we can strive towards the realization of equitable and efficacious asthma management for all patients.

## Data Availability

Due to privacy and ethical considerations, the authors can provide the dataset supporting the reported results upon request. Interested parties can contact the corresponding author directly via email to request access.
